# Comparative Analysis of Frailty Scores for Predicting Adverse Outcomes in Hip Fracture Patients: Insights from the United States National Inpatient Sample

**DOI:** 10.3390/jpm14060621

**Published:** 2024-06-10

**Authors:** Maximilian Peter Forssten, Yang Cao, Ahmad Mohammad Ismail, Lakshika Tennakoon, David A. Spain, Shahin Mohseni

**Affiliations:** 1 Department of Orthopedic Surgery, Faculty of Medicine and Health, Orebro University, Orebro 701 82, Sweden; 2 School of Medical Sciences, Orebro University, 701 82 Orebro, Sweden; amzenik@gmail.com; 3 Clinical Epidemiology and Biostatistics, School of Medical Sciences, Faculty of Medicine and Health, Orebro University, 701 82 Orebro, Sweden; yang.cao@oru.se; 4 Department of Surgery, Section of Trauma and Acute Care Surgery, Stanford University School of Medicine, Stanford, CA 94305, USA; lakshika@stanford.edu (L.T.); dspain@stanford.edu (D.A.S.); 5 Division of Trauma, Critical Care & Acute Care Surgery, Department of Surgery, Sheikh Shakhbout Medical City, Abu Dhabi P.O. Box 11001, United Arab Emirates

**Keywords:** hip fracture, frailty, mortality, morbidity, prediction, logistic regression, Orthopedic Frailty Score, Nottingham Hip Fracture Score, Modified Frailty Index, Johns Hopkins Frailty Indicator

## Abstract

The aim of the current investigation was to compare the ability of several frailty scores to predict adverse outcomes in hip fracture patients. All adult patients (18 years or older) who suffered a hip fracture due to a fall and underwent surgical fixation were extracted from the 2019 National Inpatient Sample (NIS) Database. A combination of logistic regression and bootstrapping was used to compare the predictive ability of the Orthopedic Frailty Score (OFS), the Nottingham Hip Fracture Score (NHFS), the 11-factor modified Frailty Index (11-mFI) and 5-factor (5-mFI) modified Frailty Index, as well as the Johns Hopkins Frailty Indicator. A total of 227,850 patients were extracted from the NIS. In the prediction of in-hospital mortality and failure-to-rescue (FTR), the OFS surpassed all other frailty measures, approaching an acceptable predictive ability for mortality [AUC (95% CI): 0.69 (0.67–0.72)] and achieving an acceptable predictive ability for FTR [AUC (95% CI): 0.70 (0.67–0.72)]. The NHFS demonstrated the highest predictive ability for predicting any complication [AUC (95% CI): 0.62 (0.62–0.63)]. The 11-mFI exhibited the highest predictive ability for cardiovascular complications [AUC (95% CI): 0.66 (0.64–0.67)] and the NHFS achieved the highest predictive ability for delirium [AUC (95% CI): 0.69 (0.68–0.70)]. No score succeeded in effectively predicting venous thromboembolism or infections. In summary, the investigated frailty scores were most effective in predicting in-hospital mortality and failure-to-rescue; however, they struggled to predict complications.

## 1. Introduction

Hip fractures represent a significant and growing public health concern, which predominantly afflicts the elderly population [1]. Projections indicate a substantial rise in the annual number of hip fractures, which are expected to reach 4.5 million globally by the year 2050 [2]. This is of particular concern given that the hip fracture rates in the United States are among the highest in the world [3]. With one-year postoperative mortality rates reaching 27% [4], and even exceeding 50% in certain demographic cohorts [5], this underscores the critical importance of addressing hip fractures comprehensively and urgently to mitigate their profound impact on individuals and healthcare systems alike.

This high mortality rate in hip fracture patients stems largely from the high prevalence of frailty within this population. Studies reveal stark contrasts, with crude 30-day and 90-day mortality rates among frail individuals reaching up to ten times higher than those among non-frail hip fracture patients [6]. Furthermore, frailty has been found to be the single most important predictor of in-hospital mortality in hip fracture patients [7]. Complicating matters, however, is the proliferation of published frailty scores, leading to uncertainty regarding their use and effectiveness. This equipoise underscores the need to identify which frailty scores perform optimally. Consequently, the current study aims to compare the ability of several frailty scores to predict adverse outcomes in hip fracture patients.

## 2. Materials and Methods

The data for the present study were sourced from the 2019 United States National Inpatient Sample (NIS). Managed by the Agency for Healthcare Research and Quality, the NIS constitutes the largest all-payer inpatient database in the United States, drawing from a 20% sample of the nation’s hospitalizations. The NIS is sampled from the State Inpatient Databases, including all inpatient data that are currently contributed to the Healthcare Cost and Utilization Project. Utilizing validated sampling algorithms integrated with discharge and survey weights, the NIS ensures accurate national estimates for a substantial 97% of all inpatient hospitalizations in the country [8]. The current investigation adhered to the transparent reporting of a multivariable prediction model for individual prognosis or diagnosis (TRIPOD) guidelines.

All individuals aged 18 years or older who had undergone emergency hip fracture surgery following a traumatic fall were included in this study. Surgical procedures involved internal fixation methods, including open reduction internal fixation or intramedullary nailing, as well as arthroplasty, both total hip arthroplasty and hemiarthroplasty. To reduce heterogeneity in the dataset, patients with head, vascular, or truncal injuries were excluded. Only 0.4% of the patients (N = 925) were missing data required for calculating the frailty scores; consequently, these patients were excluded. The identification of the previously listed variables relied on the International Classification of Diseases 10th Revision (ICD-10) codes recorded in the NIS [9]. The data retrieved included patient demographics (age, sex, and race/ethnicity), comorbidities, fracture morphology, surgical management, origin of admission, and functional ability, as well as discharge disposition and in-hospital complications [10].

### 2.1. Calculating Frailty Scores

#### 2.1.1. Orthopedic Frailty Score

The Orthopedic Frailty Score (OFS) was developed for the explicit purpose of evaluating frailty and predicting short-term mortality in individuals with hip fractures [11,12]. The OFS was calculated based on the presence of five dichotomous variables: congestive heart failure, a history of malignancy (excluding non-invasive skin cancer), institutionalization, dependence on assistance for activities of daily living (indicating a non-independent functional status), and an age of 85 years or older. Each of these variables was ascribed one point, resulting in a maximum possible score of 5 [11].

#### 2.1.2. Nottingham Hip Fracture Score

The Nottingham Hip Fracture Score (NHFS) was predominantly calculated according to the original study by Maxwell et al. [13]. Patients received 3 points if their age fell within the range of 66 to 85 years and 4 points if they were 86 years or older. Additionally, patients received 1 point if they were male, institutionalized, had a history of malignancy, or presented with two or more comorbidities. In the absence of a mini-mental test score, patients received 1 point if they had dementia, aligning with the methodology employed by previous investigations [11,14,15,16,17]. Furthermore, as admission hemoglobin is not recorded in the NIS, patients also received 1 point if they were diagnosed with any deficiency anemia or chronic blood loss anemia at the time of admission [18]. Subsequently, the maximum possible score was 10.

#### 2.1.3. 11-Factor Modified Frailty Index

The 11-factor modified Frailty Index (11-mFI) was determined based on the presence of non-independent functional status, hypertension requiring medication, chronic obstructive pulmonary disease (COPD), impaired sensorium, diabetes mellitus, previous myocardial infarction, congestive heart failure, stroke with neurologic deficit, transient ischemic attack or stroke without neurologic deficit, angina pectoris, and peripheral vascular disease [19,20]. However, pneumonia was not included in the calculation as it constituted one of the outcomes in the current analysis. Each variable present increased the score by 1, resulting in a maximum potential score of 11.

#### 2.1.4. 5-Factor Modified Frailty Index

The 5-factor modified Frailty Index (5-mFI) was determined based on the presence of COPD, diabetes mellitus, hypertension requiring medication, congestive heart failure, and non-independent functional status [21]. Each variable present increased the score by 1, resulting in a maximum potential score of 5.

#### 2.1.5. Johns Hopkins Frailty Indicator

As per the Johns Hopkins Frailty Indicator, patients were categorized as either non-frail or frail, contingent upon the identification of at least one diagnosis that defines frailty. These conditions included fall from wheelchair, fall on stairs or steps, abnormality of gait, difficulty in walking, inadequate material resources, inadequate housing, lack of housing, fecal incontinence, feeding difficulties and mismanagement, abnormal loss of weight and underweight, continuous urinary leakage, incontinence without sensory awareness, decubitus ulcer, profound visual impairment, senile dementia with delirium, senile dementia with delusional or depressive features, nutritional marasmus, or other severe protein-calorie malnutrition [22,23].

### 2.2. Statistical Analysis

Summary statistics were employed in order to describe the dataset. Age was summarized as a median and interquartile range, while the remaining categorical variables were presented as counts and percentages. The adverse outcomes investigated included in-hospital mortality, any complication, cardiovascular complications, delirium, venous thromboembolism, infection, and failure-to-rescue (FTR).

Any complication encompassed heart failure, cardiac arrest, cardiac tamponade, ventricular tachycardia, ventricular fibrillation, stroke, acute kidney injury, delirium, deep vein thrombosis, pulmonary embolism, embolism due to orthopedic device, urinary tract infection, pneumonia, wound infection, implant infection, sepsis, and decubitus ulcers. Cardiovascular complications were defined as heart failure, cardiac arrest, cardiac tamponade, ventricular tachycardia, ventricular fibrillation, or stroke. Venous thromboembolism comprised deep vein thrombosis, pulmonary embolism, or embolism due to orthopedic device. Infections included urinary tract infections, pneumonia, wound infections, implant infection, and sepsis. FTR was defined as in-hospital mortality subsequent to a complication.

To evaluate the predictive ability of all the included frailty scores for each outcome, LR models were fitted to the dataset with the outcome as the response variable and the frailty score as the predictor. The AUC (area under the receiver operating characteristic curve), as well as the sensitivity and specificity that maximized Youden’s index (sensitivity + specificity − 1), was calculated for each model, along with their respective 95% confidence intervals (CI). The CIs were estimated using 1000 bootstrap replicates; *p*-values for the differences in the AUCs were also estimated using 1000 bootstrap replicates as follows:

1. The AUCs for two frailty scores were calculated: ${AUC}_{1}$ and ${AUC}_{2}$.
2. The observed difference in the AUCs was calculated: $D_{observed}={AUC}_{1}-{AUC}_{2}$.
3. 1000 bootstrap replicates of the dataset were generated.
4. The difference in the AUCs for each replicate was calculated: $D_{1}\ldots D_{1000}$.
5. The standard error of the differences in the AUCs was calculated as the standard deviation across all replicates: $\sigma_{\bar{D}}=\sqrt{\frac{\sum_{i=1}^{1000}{\left(D_{i}-\bar{D}\right)}^{2}}{1000}}$.
6. The z-value was determined by dividing the difference in the AUCs by the standard error: Z=DobservedσD¯.
7. A two-sided *p*-value was determined based on the proportion of a normally distributed population with a z-value >Z or <−Z (where Z is positive).

These analyses were also repeated with a subgroup of patients consisting of those who were 50 years or older. Statistical significance was defined as a two-sided *p*-value < 0.05. All *p*-values were adjusted for multiple comparisons using Bonferroni correction. All analyses were performed in the software R 4.2.2 using the tidyverse, survey, parallel, and WeightedROC packages [24].

## 3. Results

A total of 227,795 estimated hip fracture patients were included in the current investigation. The median age was 81, with the majority of patients being female (68.8%) and White (84.4%). Most patients had either suffered a cervical (47.3%) or pertrochanteric (46.8%) hip fracture, with the most common method of fixation being internal fixation (63.4%) (Table 1). The most common comorbidities consisted of ischemic heart disease (25.2%), fluid or electrolyte disorders (24.9%), hypothyroidism (17.1%), congestive heart failure (16.6%), and end-stage renal disease (15.5%) (Table 2). In total, 1.3% (N = 2910) died in the hospital. In 1.1% (N = 2585) of cases, this was following a complication. A total of 35.2% (N = 80,135) of patients suffered one of the included complications (Table 3). The distribution of all outcomes across the levels of each score is presented in Supplemental Table S1.

In the prediction of both in-hospital mortality and FTR, the OFS surpassed all other frailty measures, approaching an acceptable predictive ability for mortality [AUC (95% CI): 0.69 (0.67–0.72)] and achieving an acceptable predictive ability for FTR [AUC (95% CI): 0.70 (0.67–0.72)] [25]. All scores struggled to predict complications; however, the NHFS demonstrated the highest predictive ability [AUC (95% CI): 0.62 (0.62–0.63)]. On the other hand, the 11-mFI demonstrated the highest predictive ability for cardiovascular complications [AUC (95% CI): 0.66 (0.64–0.67)] and the NHFS achieved the highest predictive ability for delirium [AUC (95% CI): 0.69 (0.68–0.70)]. No score succeeded in effectively predicting venous thromboembolism or infections (Table 4). The results remained virtually unchanged in the subgroup analysis composed of patients who were 50 years or older (Supplemental Table S2).

## 4. Discussion

By leveraging the NIS, which samples 20% of the nation’s hospitalizations and accurately estimates 97% of all inpatient hospitalizations in the United States, a nationwide analysis could be conducted comparing the predictive ability of a range of frailty scores. Among the adverse outcomes investigated, these scores functioned best when predicting mortality and failure-to-rescue. Regarding these particular outcomes, the OFS demonstrated better performance compared to all other scores, with the NHFS following in second place. However, all scores comparatively struggled to accurately predict in-hospital complications, with some exceptions for specific types of complications. Notably, the NHFS stood out in its ability to predict delirium, while the 11-mFI outperformed all other scores when predicting cardiovascular complications. Of all the scores, only the OFS and NHFS were able to demonstrate an acceptable or close-to-acceptable predictive ability for any of the adverse outcomes [25].

These findings are by and large in line with prior studies. In the original study that developed and validated the OFS [AUC (95% CI): 0.77 (0.74–0.80)], it was found to surpass the 5-mFI [AUC (95% CI): 0.69 (0.66–0.73)] in predicting mortality, which is consistent with the present investigation. Furthermore, the OFS in the current study displays essentially the same predictive ability for mortality as it did in the national dataset used for developing the OFS. However, in the original study, the NHFS [AUC (95% CI): 0.76 (0.73–0.79)] and the OFS [AUC (95% CI): 0.77 (0.74–0.80)] demonstrated comparable predictive performance. This difference could be due to the lack of an admission hemoglobin or the fact that the current investigation focused on in-hospital mortality rather than 30-day postoperative mortality [11]. An analysis utilizing the NIS to explore the relationship between dementia and frailty also found that the OFS was the most important predictor of in-hospital mortality among the 52 variables analyzed, providing further support for its robust predictive capability in comparison to other scoring systems [7]. In a study conducted by Thorne et al., the AUC for in-hospital mortality was reported as 0.69 (95% CI: 0.64–0.74) for the NHFS, slightly higher than the NHFS’s performance and on the same level as the OFS observed in the current investigation [26]. A separate analysis conducted by Lisk et al. reported an AUC for in-hospital mortality of 0.674 (95% CI: 0.584–0.764) for the NHFS; however, the wide confidence interval makes it difficult to judge if this indicates a true difference compared to the NHFS and the OFS in the current study [27]. Finally, when investigating the predictive performance of the 5-mFI, Ondeck et al. calculated an AUC for in-hospital mortality of 0.641 (95% CI: 0.624–0.659), which was higher than the current analysis. Nevertheless, similar to the current findings, the 5-mFI struggled to predict complications, with an AUC of 0.586 (95% CI: 0.581–0.592) for all adverse events [28].

As the current analyses were limited to the variables available in the NIS, there are many frailty scores that could not be included in the current analysis but warrant acknowledgment. In the same investigation by Thorne et al. that examined the NHFS, they also found that the Clinical Frailty Scale (CFS) yielded an AUC of 0.63 (95% CI: 0.57–0.69) for in-patient mortality, indicating a lower predictive ability compared to both the NHFS and the OFS [26]. In another study by Choi et al., the hip-multidimensional frailty score (Hip-MFS) exhibited a significantly better ability to predict postoperative complications with an AUC of 0.679 (95% CI: 0.613–0.745), surpassing all scores encompassed in the current study [29]. Additionally, the Hospital Frailty Risk Score (HFRS), composed of over 100 weighted variables, has demonstrated associations with heightened risks of in-hospital mortality, complications, prolonged length of stay, and increased total hospital costs [30,31]. Similarly, the FRAIL scale has been linked to elevated risks of postoperative complications and extended length of stay in geriatric fracture patients [32]. Employing a modified version of the Fried Frailty Index, Kistler et al. found that it was associated with an increased risk of complications [33]. Meanwhile, the Frailty Index, comprising over 50 variables, was associated with an elevated mortality rate and longer hospital stays [34].

Despite the wide range of frailty scores available, there remains a clinical equipoise when selecting which to apply in practice. Many feature an extensive variable list, which may hinder their ability to be used bedside [30,31,34,35]. Additionally, some also require patient cooperation [32,33,35], which may be challenging in a patient population where approximately 20% suffer from dementia [36]. Moreover, the need for blood tests post-admission further complicates the use of others [13,35]. Finally, some scores are inherently more subjective, relying on clinicians’ judgment and observation of functional abilities in conjunction with overall health [37]. This in turn results in greater interobserver variation, especially among inexperienced users [38,39,40,41]. Despite the intuitive advantages of complexity, the current findings suggest that more intricate indices are not inherently superior. Notably, the OFS, among the simplest of the included scores, emerges as particularly adept at predicting mortality-related outcomes. Furthermore, the mFI-5, which requires fewer than half as many variables as the mFI-11, generally exhibits only marginal differences compared to the mFI-11, with the prediction of cardiovascular complications being the only exception. Additionally, research indicates that orthopedic surgeons tend to favor simpler and more straightforward scoring systems for clinical application [42]; this is an important point to consider when selecting a tool to transition from research settings to clinical practice.

This study was strengthened by the use of the NIS, which constitutes the largest all-payer inpatient database in the United States [8]. Leveraging this extensive dataset enabled the investigation of a wide array of outcomes through the inclusion of over 227,000 estimated hip fracture patients. However, a notable limitation was the absence of admission hemoglobin data, necessitating reliance on the diagnosis of anemia, which could potentially have reduced the predictive ability of the NHFS. Nonetheless, despite this alteration, the NHFS still outperformed all other scores in predicting delirium by a significant margin. Furthermore, our analyses were constrained by the variables available in the NIS, preventing the inclusion of other pertinent outcomes such as readmission, quality of life, and long-term mortality. Additionally, the risk of non-differential misclassification remains a concern when using a retrospective database, as this adds statistical noise to the data that reduces the overall predictive ability of all scores.

## 5. Conclusions

The investigated frailty scores were most effective in predicting in-hospital mortality and failure-to-rescue; however, they struggled to predict complications. The findings suggest that increasing complexity in frailty scores may result in diminishing returns concerning predictive ability, as evidenced by the Orthopedic Frailty Score, which, despite its simplicity in calculation, either outperforms or performs comparably to the other scores across most outcomes. Future studies should investigate how these scores can be incorporated into routine preoperative evaluations to guide care through preoperative optimization and resource allocation.

## Figures and Tables

**Table 1:** Demographics of hip fracture patients.

	All Patients (N = 227,795)
Age, median [IQR]	81 [72.0–88.0]
Sex, n (%)	
Male	71,050 (31.2)
Female	156,745 (68.8)
Race/Ethnicity, n (%)	
White	192,245 (84.4)
Black	9230 (4.1)
Hispanic	12,235 (5.4)
Asian or Pacific Islander	3950 (1.7)
American Indian	975 (0.4)
Other	4405 (1.9)
Missing	4755 (2.1)
Type of fracture, n (%)	
Cervical	107,640 (47.3)
Basicervical	3770 (1.7)
Pertrochanteric	106,625 (46.8)
Subtrochanteric	9655 (4.2)
Missing	105 (0.0)
Type of surgery, n (%)	
Internal fixation	144,425 (63.4)
Arthroplasty	83,370 (36.6)
OFS, n (%)	
0	108,580 (47.7)
1	87,215 (38.3)
2	28,315 (12.4)
3	3420 (1.5)
4	265 (0.1)
NHFS, n (%)	
0	5525 (2.4)
1	12,940 (5.7)
2	7825 (3.4)
3	22,885 (10.0)
4	56,285 (24.7)
5	62,540 (27.5)
6	41,895 (18.4)
7	15,010 (6.6)
8	2655 (1.2)
9	230 (0.1)
10	5 (0.0)
11-mFI, n (%)	
0	28,390 (12.5)
1	65,070 (28.6)
2	68,060 (29.9)
3	41,230 (18.1)
4	18,345 (8.1)
5	5585 (2.5)
6	1025 (0.4)
7	85 (0.0)
8	5 (0.0)
5-mFI, n (%)	
0	46,430 (20.4)
1	98,260 (43.1)
2	61,855 (27.2)
3	18,215 (8.0)
4	2870 (1.3)
5	165 (0.1)
Johns Hopkins Frailty Indicator, n (%)	
0	164,200 (72.1)
1	63,595 (27.9)

OFS, Orthopedic Frailty Score; NHFS, Nottingham Hip Fracture Score; 11-mFI, 11-factor modified Frailty Index; 5-mFI, 5-factor modified Frailty Index.

**Table 2:** Comorbidities in hip fracture patients.

	All Patients (N = 227,795)
Ischemic heart disease, n (%)	57,360 (25.2)
Congestive heart failure, n (%)	37,900 (16.6)
Cerebrovascular disease, n (%)	14,280 (6.3)
Peripheral vascular disease, n (%)	10,610 (4.7)
Diabetes mellitus without complications, n (%)	20,315 (8.9)
Diabetes mellitus with complications, n (%)	27,270 (12.0)
End stage renal disease, n (%)	35,385 (15.5)
Liver disease, n (%)	4500 (2.0)
Deficiency anemia, n (%)	32,780 (14.6)
Chronic blood loss anemia, n (%)	2545 (1.1)
Coagulopathy, n (%)	12,395 (5.4)
Obesity, n (%)	10,075 (4.4)
Alcohol abuse, n (%)	7650 (3.4)
Drug abuse, n (%)	3010 (1.3)
Psychosis, n (%)	5805 (2.5)
Depression, n (%)	25,275 (11.1)
Current smoker, n (%)	1455 (0.6)
COPD, n (%)	42,035 (18.5)
Dementia, n (%)	58,170 (25.5)
Impaired sensorium, n (%)	66,585 (29.2)
Non-independent functional status, n (%)	10,795 (4.7)
Institutionalized, n (%)	12,405 (5.4)
Osteoporosis, n (%)	34,790 (15.3)
Osteoporosis with fracture, n (%)	2335 (1.0)
Fluid or electrolyte disorder, n (%)	56,650 (24.9)
Connective tissue disease, n (%)	7365 (3.2)
Hypothyroidism, n (%)	39,010 (17.1)
Local cancer, n (%)	4090 (1.8)
Metastatic cancer, n (%)	2465 (1.1)
Leukemia, n (%)	1405 (0.6)
Lymphoma, n (%)	1620 (0.7)

COPD, Chronic obstructive pulmonary disease.

**Table 3:** Crude outcomes in hip fracture patients.

	All Patients (N = 227,795)
In-hospital mortality, n (%)	2910 (1.3)
Complication, n (%)	80,135 (35.2)
Any cardiac complication	3660 (1.6)
Heart failure	505 (0.2)
Cardiac arrest	185 (0.1)
Cardiac tamponade	20 (0.0)
Ventricular tachycardia	2285 (1.0)
Ventricular fibrillation	190 (0.1)
Stroke	2160 (0.9)
Acute kidney injury	34,355 (15.1)
Delirium	8970 (3.9)
Deep vein thrombosis	1545 (0.7)
Pulmonary embolism	1240 (0.5)
Embolism due to orthopedic device	10 (0.0)
Urinary tract infection	26,680 (11.7)
Pneumonia	21,590 (9.5)
Wound infection	160 (0.1)
Implant infection	30 (0.0)
Sepsis	3995 (1.8)
Decubitus ulcer	3955 (1.7)
Failure-to-rescue, n (%)	2615 (1.1)

**Table 4:** Predictive ability of frailty scores for adverse outcomes in hip fracture patients.

Outcome	AUC (95% CI)	Sensitivity (95% CI)	Specificity (95% CI)	*p*-Value for Difference in AUCs
In-hospital mortality				
OFS	0.69 (0.67–0.72)	0.83 (0.80–0.86)	0.48 (0.48–0.49)	Reference
NHFS	0.65 (0.63–0.67)	0.76 (0.74–0.82)	0.47 (0.43–0.47)	<0.001
11-mFI	0.61 (0.59–0.64)	0.45 (0.42–0.75)	0.71 (0.41–0.72)	<0.001
5-mFI	0.57 (0.55–0.59)	0.47 (0.18–0.68)	0.64 (0.43–0.91)	<0.001
Johns Hopkins Frailty Indicator	0.56 (0.54–0.58)	0.39 (0.35–0.43)	0.72 (0.72–0.73)	<0.001
Any complication				
OFS	0.60 (0.60–0.61)	0.64 (0.63–0.65)	0.54 (0.53–0.55)	Reference
NHFS	0.62 (0.62–0.63)	0.66 (0.65–0.66)	0.53 (0.52–0.53)	<0.001
11-mFI	0.61 (0.60–0.62)	0.69 (0.69–0.70)	0.47 (0.46–0.47)	0.147
5-mFI	0.57 (0.57–0.58)	0.44 (0.43–0.45)	0.68 (0.67–0.68)	<0.001
Johns Hopkins Frailty Indicator	0.55 (0.55–0.56)	0.35 (0.34–0.36)	0.76 (0.75–0.76)	<0.001
Cardiovascular complication				
OFS	0.60 (0.59–0.62)	0.69 (0.66–0.72)	0.48 (0.48–0.49)	Reference
NHFS	0.60 (0.58–0.61)	0.72 (0.62–0.74)	0.43 (0.43–0.54)	1.00
11-mFI	0.66 (0.64–0.67)	0.53 (0.50–0.56)	0.72 (0.71–0.72)	<0.001
5-mFI	0.56 (0.54–0.57)	0.46 (0.17–0.48)	0.64 (0.63–0.91)	<0.001
Johns Hopkins Frailty Indicator	0.53 (0.51–0.54)	0.33 (0.30–0.36)	0.72 (0.72–0.73)	<0.001
Delirium				
OFS	0.61 (0.60–0.63)	0.72 (0.70–0.74)	0.48 (0.48–0.49)	Reference
NHFS	0.69 (0.68–0.70)	0.81 (0.79–0.83)	0.47 (0.47–0.48)	<0.001
11-mFI	0.59 (0.58–0.61)	0.74 (0.72–0.76)	0.42 (0.41–0.42)	0.021
5-mFI	0.51 (0.51–0.53)	0.74 (0.29–0.77)	0.29 (0.27–0.72)	<0.001
Johns Hopkins Frailty Indicator	0.58 (0.57–0.59)	0.44 (0.41–0.46)	0.73 (0.72–0.73)	<0.001
Venous thromboembolism				
OFS	0.54 (0.52–0.57)	0.60 (0.19–0.64)	0.48 (0.47–0.86)	Reference
NHFS	0.54 (0.53–0.57)	0.43 (0.33–0.79)	0.62 (0.27–0.72)	1.00
11-mFI	0.53 (0.52–0.56)	0.46 (0.34–0.75)	0.59 (0.32–0.71)	1.00
5-mFI	0.54 (0.53–0.57)	0.35 (0.25–0.66)	0.72 (0.43–0.80)	1.00
Johns Hopkins Frailty Indicator	0.54 (0.52–0.56)	0.35 (0.31–0.39)	0.72 (0.72–0.73)	1.00
Infection				
OFS	0.58 (0.58–0.59)	0.63 (0.62–0.64)	0.50 (0.50–0.51)	Reference
NHFS	0.58 (0.57–0.58)	0.63 (0.62–0.86)	0.49 (0.24–0.49)	0.764
11-mFI	0.58 (0.58–0.59)	0.69 (0.39–0.70)	0.44 (0.43–0.73)	1.00
5-mFI	0.56 (0.55–0.56)	0.44 (0.43–0.45)	0.65 (0.65–0.66)	<0.001
Johns Hopkins Frailty Indicator	0.54 (0.54–0.55)	0.34 (0.33–0.35)	0.74 (0.73–0.74)	<0.001
Failure-to-rescue				
OFS	0.70 (0.67–0.72)	0.83 (0.80–0.87)	0.48 (0.48–0.49)	Reference
NHFS	0.65 (0.63–0.67)	0.77 (0.75–0.83)	0.47 (0.43–0.47)	<0.001
11-mFI	0.61 (0.59–0.64)	0.46 (0.42–0.75)	0.71 (0.41–0.72)	<0.001
5-mFI	0.57 (0.55–0.60)	0.48 (0.19–0.70)	0.64 (0.43–0.91)	<0.001
Johns Hopkins Frailty Indicator	0.56 (0.54–0.58)	0.39 (0.35–0.43)	0.72 (0.72–0.73)	<0.001

AUC, area under the receiver operating characteristic curve; OFS, Orthopedic Frailty Score; NHFS, Nottingham Hip Fracture Score; 11-mFI, 11-factor modified Frailty Index; 5-mFI, 5-factor modified Frailty Index.

## Data Availability

Data is available upon reasonable request.

## Supplementary Materials

The following supporting information can be downloaded at https://www.mdpi.com/article/10.3390/jpm14060621/s1: Table S1: Distribution of outcomes for each frailty score; Table S2: Predictive ability of frailty scores for adverse outcomes in hip fracture patients who are ≥50 years old.
