# Supplementary material for: Comparative Analysis of Frailty Scores for Predicting Adverse Outcomes in Hip Fracture Patients: Insights from the United States National Inpatient Sample
Source: J Pers Med. 2024 Jun 10;14(6):621. doi: 10.3390/jpm14060621 (PMC11204756; doi:10.3390/jpm14060621)
Supplement: Supplementary file 1 [file jpm-14-00621-s001.zip › jpm-3021772-supplementary.pdf]

Supplemental Table S1. Distribution of outcomes for each frailty score

	In-hospital mortality (N = 2,910)	Any Complication (N = 80,135)	Cardiovascular complication (N = 5,685)	Delirium (N = 8,970)	VTE (N = 2,550)	Infection (N = 46,055)	FTR (N = 2,615)
**OFS, n (%)**							
0	495 (0.5)	28,815 (26.5)	1,745 (1.6)	2,530 (2.3)	1,030 (0.9)	16,925 (15.6)	440 (0.4)
1	1,345 (1.5)	34,770 (39.9)	2,530 (2.9)	4,305 (4.9)	1,045 (1.2)	19,555 (22.4)	1,205 (1.4)
2	925 (3.3)	14,515 (51.3)	1,240 (4.4)	1,860 (6.6)	415 (1.5)	8,295 (29.3)	825 (2.9)
3	125 (3.7)	1,900 (55.6)	165 (4.8)	235 (6.9)	55 (1.6)	1,190 (34.8)	125 (3.7)
4	20 (7.5)	135 (50.9)	5 (1.9)	40 (15.1)	5 (1.9)	90 (34.0)	20 (7.5)
**NHFS, n (%)**							
0	0 (0.0)	550 (10.0)	20 (0.4)	0 (0.0)	30 (0.5)	415 (7.5)	0 (0.0)
1	45 (0.3)	2,655 (20.5)	115 (0.9)	50 (0.4)	130 (1.0)	1,805 (13.9)	40 (0.3)
2	90 (1.2)	2,380 (30.4)	200 (2.6)	90 (1.2)	135 (1.7)	1,235 (15.8)	85 (1.1)
3	70 (0.3)	4,430 (19.4)	315 (1.4)	270 (1.2)	240 (1.0)	2,740 (12.0)	55 (0.2)
4	480 (0.9)	17,535 (31.2)	1,120 (2.0)	1,250 (2.2)	610 (1.1)	11,030 (19.6)	415 (0.7)
5	965 (1.5)	24,415 (39.0)	1,950 (3.1)	2,660 (4.3)	765 (1.2)	13,655 (21.8)	870 (1.4)
6	835 (2.0)	19,355 (46.2)	1,455 (3.5)	3,075 (7.3)	425 (1.0)	10,715 (25.6)	775 (1.8)
7	355 (2.4)	7,380 (49.2)	425 (2.8)	1,300 (8.7)	185 (1.2)	3,725 (24.8)	305 (2.0)
8	65 (2.4)	1,320 (49.7)	70 (2.6)	240 (9.0)	25 (0.9)	680 (25.6)	65 (2.4)
9	5 (2.2)	115 (50.0)	15 (6.5)	35 (15.2)	5 (2.2)	55 (23.9)	5 (2.2)
10	0 (0.0)	0 (0.0)	0 (0.0)	0 (0.0)	0 (0.0)	0 (0.0)	0 (0.0)
**11-mFI, n (%)**							
0	190 (0.7)	5,485 (19.3)	240 (0.8)	475 (1.7)	320 (1.1)	3,350 (11.8)	170 (0.6)
1	595 (0.9)	19,010 (29.2)	965 (1.5)	1,885 (2.9)	830 (1.3)	11,035 (17.0)	535 (0.8)
2	805 (1.2)	24,885 (36.6)	1,465 (2.2)	3,095 (4.5)	675 (1.0)	14,100 (20.7)	710 (1.0)
3	650 (1.6)	18,045 (43.8)	1,545 (3.7)	2,100 (5.1)	460 (1.1)	10,420 (25.3)	595 (1.4)
4	465 (2.5)	9,225 (50.3)	995 (5.4)	990 (5.4)	205 (1.1)	5,130 (28.0)	425 (2.3)
5	160 (2.9)	2,930 (52.5)	390 (7.0)	340 (6.1)	45 (0.8)	1,725 (30.9)	145 (2.6)
6	40 (3.9)	490 (47.8)	80 (7.8)	70 (6.8)	15 (1.5)	265 (25.9)	30 (2.9)
7	5 (5.9)	65 (76.5)	5 (5.9)	15 (17.6)	0 (0.0)	30 (35.3)	5 (5.9)
8	0 (0.0)	0 (0.0)	0 (0.0)	0 (0.0)	0 (0.0)	0 (0.0)	0 (0.0)

**5-mFI, n (%)**							
0	545 (1.2)	13,035 (28.1)	975 (2.1)	1,895 (4.1)	685 (1.5)	7,760 (16.7)	495 (1.1)
1	1,000 (1.0)	31,865 (32.4)	2,120 (2.2)	3,935 (4.0)	975 (1.0)	17,980 (18.3)	875 (0.9)
2	830 (1.3)	24,435 (39.5)	1,680 (2.7)	2,245 (3.6)	650 (1.1)	14,015 (22.7)	770 (1.2)
3	430 (2.4)	9,300 (51.1)	770 (4.2)	780 (4.3)	215 (1.2)	5,410 (29.7)	395 (2.2)
4	90 (3.1)	1,415 (49.3)	125 (4.4)	105 (3.7)	25 (0.9)	835 (29.1)	65 (2.3)
5	15 (9.1)	85 (51.5)	15 (9.1)	10 (6.1)	0 (0.0)	55 (33.3)	15 (9.1)
**JHFI, n (%)**							
0	1,775 (1.1)	52,105 (31.7)	3,810 (2.3)	5,050 (3.1)	1,650 (1.0)	30,210 (18.4)	1,590 (1.0)
1	1,135 (1.8)	28,030 (44.1)	1,875 (2.9)	3,920 (6.2)	900 (1.4)	15,845 (24.9)	1,025 (1.6)

VTE, Venous thromboembolism; FTR, Failure-to-rescue; OFS, Orthopedic Frailty Score; NHFS, Nottingham Hip Fracture Score; 11-mFI, 11-factor modified Frailty Index; 5-mFI, 5-factor modified Frailty Index; JHFI, Johns Hopkins Frailty Indicator

Supplemental Table S2. Predictive ability of frailty scores for adverse outcomes in hip fracture patients who are ≥50 years old

Outcome	AUC (95% CI)	Sensitivity (95% CI)	Specificity (95% CI)	P-value for difference in AUCs
**In-hospital mortality**				
OFS	0.69 (0.67-0.71)	0.83 (0.80-0.86)	0.47 (0.47-0.48)	Reference
NHFS	0.64 (0.63-0.67)	0.77 (0.74-0.82)	0.46 (0.42-0.46)	<0.001
11-mFI	0.61 (0.59-0.63)	0.46 (0.42-0.76)	0.71 (0.40-0.71)	<0.001
5-mFI	0.57 (0.55-0.59)	0.47 (0.18-0.69)	0.63 (0.43-0.91)	<0.001
Johns Hopkins Frailty Indicator	0.56 (0.54-0.57)	0.39 (0.35-0.43)	0.72 (0.72-0.73)	<0.001
**Any complication**				
OFS	0.60 (0.60-0.61)	0.64 (0.64-0.65)	0.53 (0.53-0.54)	Reference
NHFS	0.62 (0.61-0.62)	0.66 (0.65-0.67)	0.52 (0.51-0.52)	<0.001
11-mFI	0.61 (0.60-0.61)	0.70 (0.69-0.70)	0.46 (0.45-0.46)	0.467
5-mFI	0.57 (0.56-0.57)	0.44 (0.43-0.45)	0.67 (0.67-0.68)	<0.001
Johns Hopkins Frailty Indicator	0.55 (0.55-0.56)	0.35 (0.34-0.36)	0.76 (0.75-0.76)	<0.001
**Cardiovascular complication**				
OFS	0.60 (0.59-0.62)	0.69 (0.67-0.72)	0.48 (0.47-0.48)	Reference
NHFS	0.59 (0.58-0.61)	0.73 (0.63-0.75)	0.43 (0.42-0.53)	1.00
11-mFI	0.66 (0.64-0.67)	0.53 (0.51-0.56)	0.71 (0.71-0.72)	<0.001
5-mFI	0.55 (0.54-0.57)	0.46 (0.17-0.49)	0.63 (0.63-0.91)	<0.001
Johns Hopkins Frailty Indicator	0.53 (0.51-0.54)	0.33 (0.30-0.36)	0.72 (0.72-0.73)	<0.001
**Delirium**				
OFS	0.61 (0.60-0.62)	0.72 (0.70-0.74)	0.48 (0.47-0.48)	Reference
NHFS	0.69 (0.68-0.70)	0.82 (0.54-0.83)	0.47 (0.46-0.74)	<0.001
11-mFI	0.59 (0.58-0.60)	0.74 (0.72-0.76)	0.41 (0.41-0.42)	0.022
5-mFI	0.52 (0.51-0.53)	0.74 (0.24-0.76)	0.29 (0.27-0.79)	<0.001
Johns Hopkins Frailty Indicator	0.58 (0.57-0.59)	0.44 (0.41-0.46)	0.73 (0.72-0.73)	0.001
**Venous thromboembolism**				
OFS	0.54 (0.52-0.57)	0.60 (0.18-0.64)	0.47 (0.47-0.86)	Reference
NHFS	0.53 (0.53-0.57)	0.43 (0.27-0.80)	0.62 (0.26-0.79)	1.00
11-mFI	0.53 (0.52-0.56)	0.45 (0.35-0.75)	0.59 (0.33-0.71)	1.00
5-mFI	0.54 (0.52-0.57)	0.35 (0.25-0.65)	0.72 (0.44-0.81)	1.00
Johns Hopkins Frailty Indicator	0.54 (0.52-0.56)	0.36 (0.32-0.40)	0.72 (0.72-0.72)	1.00

**Infection**				
OFS	0.58 (0.57-0.59)	0.63 (0.63-0.64)	0.50 (0.49-0.50)	Reference
NHFS	0.57 (0.57-0.58)	0.63 (0.62-0.64)	0.48 (0.47-0.48)	0.258
11-mFI	0.58 (0.58-0.59)	0.69 (0.38-0.70)	0.43 (0.42-0.73)	1.00
5-mFI	0.56 (0.55-0.56)	0.44 (0.43-0.45)	0.65 (0.65-0.66)	<0.001
Johns Hopkins Frailty Indicator	0.54 (0.54-0.55)	0.34 (0.34-0.35)	0.74 (0.73-0.74)	<0.001
**Failure-to-rescue**				
OFS	0.69 (0.67-0.71)	0.83 (0.80-0.86)	0.47 (0.47-0.48)	Reference
NHFS	0.65 (0.63-0.67)	0.77 (0.75-0.83)	0.46 (0.42-0.46)	<0.001
11-mFI	0.61 (0.59-0.63)	0.46 (0.42-0.76)	0.71 (0.41-0.71)	<0.001
5-mFI	0.57 (0.55-0.60)	0.48 (0.19-0.70)	0.63 (0.43-0.91)	<0.001
Johns Hopkins Frailty Indicator	0.56 (0.54-0.58)	0.39 (0.35-0.43)	0.72 (0.72-0.73)	<0.001

OFS, Orthopedic Frailty Score; NHFS, Nottingham Hip Fracture Score; 11-mFI, 11-factor modified Frailty Index; 5-mFI, 5-factor modified Frailty Index
